# Editorial: Quantum and quantum-like effects across neuroscience

**DOI:** 10.3389/fnhum.2026.1838245

**Published:** 2026-04-08

**Authors:** Michael C. Wiest, Daya Shankar Gupta

**Affiliations:** 1 Neuroscience Department, Wellesley College, Wellesley, MA, United States; 2 Neuroscience Department, South University, Savannah, GA, United States

**Keywords:** active inference, consciousness, contextuality, entanglement, microtubules (MTs), orchestrated objective reduction (Orch OR), quantum cognition, quantum like models

For about 30 years now a growing literature has applied quantum probability theory to successfully describe a wide range of statistical question-answering phenomena in psychology that appear irrational from a classical perspective (Pothos and Busemeyer, 2013, 2022; Bruza et al., 2009, 2015; Atmanspacher et al., 2004; Aerts and Aerts, 1995; Huang et al., 2025; Wang et al., 2014; Basieva et al., 2019; Adler, 2003; Khrennikov, 2023; Tsuchiya et al., 2025; Cervantes and Dzhafarov, 2019).

In the present volume, Whittle-Walls presents a systematic motivation for applying a quantum-like (QL) statistical framework to model human behavior “beyond individual cognition,” at the level of networks of reasoning individuals and social institutions. That study also provides a generic task-independent decomposition of QL effects into several clearly delineated phenomena whose experimental signatures can be compared across domains such as psychology, political science, sociology, and neuroscience. This paper can also function as a concise introduction to QL modeling of cognition, with concrete examples showing how human choice behaviors treated as errors or *ad hoc* special cases under a classical formalism, can be understood as *systematic context-sensitivity* under a QL model.

Lawless in the present volume uses a comparable approach to interpret public data pertaining to the effectiveness of teams, arguing that the QL description reveals performance gains from allowing “interdependence” among team members, that are obscured under a purely classical model. Interdependence here can be understood as an aspect of the context-sensitivity inherent in QL models.

The behavioral results of the QL cognition program are striking and robust enough (Basieva et al., 2019; Wang et al., 2014; Huang et al., 2025) that researchers have begun to consider potential (classical) neural implementations for generating QL cognition (Busemeyer et al., 2017). Testing neural models for generating QL cognition will require *neural* measures of context-sensitivity, which is the focus of the study by Khrennikov and Yamada in the present volume. That paper formulates a model of QL decision-making in terms of oscillatory states of neural networks comparable to those modeled by

Busemeyer et al. (2017). The authors derive rigorous measures of “mental entanglement” expressing the statistical inseparability—i.e., interdependence—of neural variables that represent cognitive variables, and discuss how these measures could be extracted from EEG or MEG data. Excitingly, applying these measures to directly test for cognitive entanglement in EEG/MEG data during task performance “remains an open direction for future research.”

Does mental entanglement or “true contextuality” (Basieva et al., 2019; Bruza et al., 2023; Cervantes and Dzhafarov, 2019) imply a genuinely quantum physical implementation in the brain? Not necessarily. A number of authors have argued that some quantum-like statistical phenomena, such as incompatible observables and interference, can emerge in complex classical systems when information is coarse-grained or otherwise inaccessible (Graben and Atmanspacher, 2006; De Barros and Suppes, 2009; Stewart et al., 2011; Stewart and Eliasmith, 2013). However, we are not aware of a realistic classical neural model exhibiting true contextuality as defined in Basieva et al. (2019), Bruza et al. (2023), and Cervantes and Dzhafarov (2019).

The idea that quantum physics might be relevant to understanding how the brain generates conscious cognition has been considered laughable since it was proposed by Nobel Laureate Roger Penrose and others (Hameroff and Penrose, 1996, 2014) in the 1990s. The main objection was that the brain is too wet and *warm* to sustain functional quantum effects. This objection (Tegmark, 2000) was never conclusive (Hagan et al., 2002), but in recent years evidence has accumulated from multiple sources to support the physical plausibility of the quantum consciousness hypothesis, particularly with respect to microtubules (MTs), the cytoskeletal proteins hypothesized by Hameroff and Penrose (1996) and Hameroff and Penrose (2014) to support consciousness. These include experimental reports from independent labs, suggesting quantum physical effects in MTs *at room temperature, in vitro* (Babcock et al., 2024) and in living neurons (Saxena et al., 2020; Singh et al., 2021a,b). Familiar local field potential oscillations have been shown to be *driven* by MT resonances in the honeybee brain (Gutierrez et al., 2021). Other work implicates MTs (Linganna et al., 2015; Craddock et al., 2017; Kalra et al., 2023; Huang et al., 2026; Khan et al., 2024; Yu et al., 2026) and quantum spin (Li et al., 2018; Turin et al., 2014) in anesthetic mechanisms, and non-classical MRI signals appear to support quantum consciousness in living humans (Kerskens and Pérez, 2022; Lopez Perez et al., 2023). A quantum substrate of consciousness has also been argued to solve otherwise intractable conceptual problems such as the phenomenal binding problem and the problem of accounting for the evolution of adaptive conscious states (Georgiev, 2023; Wiest, 2025). From the cognitive perspective, it has been pointed out that objective quantum wavefunction collapse dynamics on MTs provide a natural *implementation* (Wiest and Puniani, 2025a,b) of the empirically well-supported active inference model of cognition (Parr et al., 2022).

Quantifying the contribution of classical and quantum mechanisms to brain function is a major challenge for neuroscience going forward, demanding rigorous quantitative frameworks for interpreting the various kinds of relevant data. Two papers in the present volume by Sergi et al. and Theise and Tuszynski address this challenge by proposing complementary frameworks for quantitatively describing classical structure, decoherence, and quantum effects in a physical brain.

Theise and Tuszynski in this volume describe the Method of Coherent Structures, which formulates how larger-scale structures provide a “classical envelope” for smaller-scale quantum processes, and allows for the incorporation of energy dissipation and pumping terms characterizing the far-from-thermodynamic-equilibrium living state. Interestingly, a cubic non-linearity arises naturally in this kind of model, which may provide support for a little-known quantum model of visual pursuit (eye-tracking) (Behera et al., 2005) that depends on a cubic non-linearity in its quantum evolution equation, and outperforms classical models.

Sergi et al. in the present volume advocate for the Quantum-Classical formalism, which also allows for the incorporation of non-equilibrium phenomena. Under this approach, the system under study is decomposed into classical and quantum subsystems. In contrast to Theise and Tuszynski, Sergi et al. focus on using the formalism to quantitatively distinguish objective wavefunction collapse predicted under the Penrose and Hameroff theory, from decoherence induced by interaction with the environment.

Although MTs are a dominant hypothesized substrate for functional quantum brain processes, a number of other possibilities have been suggested (Fisher, 2015; Jedlicka, 2017). Keppler in the present volume explores the possibility that the excitatory neurotransmitter glutamate has appropriate physical properties to couple collectively to the quantum ground state of the brain's electromagnetic field, also known as the zero-point field or quantum vacuum. Rather than nothingness, this “vacuum” could actually represent the background structure or “envelope” described in Sergi et al. and Theise and Tuszynski. Thus, the glutamate proposal of Keppler appears compatible with the MT hypothesis discussed above.

Collectively, the articles in this volume present tools for evaluating competing classical and quantum models of cognition at the behavioral, neural, and biophysical levels. We hope they will foster wider discussion of these issues in the neuropsychological community, and catalyze focused experimental tests.
